# Supplementary material for: Dynalign II: common secondary structure prediction for RNA homologs with domain insertions
Source: Nucleic Acids Res. 2014 Nov 21;42(22):13939–48. doi: 10.1093/nar/gku1172 (PMC4267632; doi:10.1093/nar/gku1172)
Supplement: SUPPLEMENTARY DATA [file supp_gku1172_nar-02021-z-2014-File012.zip › manual/GUI/html/Refold.html]

RNAstructure GUI Help -- Refold


|  |  |  |
| --- | --- | --- |
|  | RNAstructure GUI Help Refold | - Contents - Index |
| **Refolding Previously Folded Structures**  The refold module, available from the menu option "File -> Refold From Save File," generates a new set of suboptimal secondary structures from a folding save file. This is much faster than predicting the first set of suboptimal secondary structures. The refold module works for both RNA or DNA sequences because the energy parameters are stored in the SAVE file.  The same three parameters that apply to folding (Maximum Percent Energy Difference, Maximum Number of Structures, Window Size) also apply to refolding. See "Fold."  During refolding, the temperature of folding cannot be changed, and new constraints cannot be imposed on refolding. The constraints previously applied can be displayed by choosing "Force -> Show Current Constraints."   ---  **Refolding Dynalign Calculations**   1. Choose "File -> Refold From Dynalign Save File" to open the module. 2. Choose the Dynalign save file by clicking the "Save File" button. 3. Choose an output file for the structures in the first sequence by clicking the "CT File 1" button. 4. Choose an output file for the structures in the second sequence by clicking the "CT File 2" button. 5. Choose an output file for the alignment by clicking the "Alignment File" button. 6. The default values for generation of suboptimal structures can be changed, if desired. 7. Folding constraints are set during the original calculation, but these settings can be reviewed by choosing "Force -> Show Current Constraints." 8. Click "Start." When the calculation is complete, the option to display the structures will be offered. Click "OK" to view the structures or "Cancel" to close the module. | | |
| Visit The Mathews Lab RNAstructure Page for updates and latest information. | | |
